# Impact of different interventions on preventing suicide and suicide attempt among children and adolescents in the United States: a microsimulation model study

**DOI:** 10.3389/fpsyt.2023.1127852

**Published:** 2023-06-02

**Authors:** Chengchen Zhang, Zafar Zafari, Julia F. Slejko, Wendy Camelo Castillo, Gloria M. Reeves, Susan dosReis

**Affiliations:** ^1^Shanghai Children's Medical Center Affiliated With Shanghai Jiao Tong University School of Medicine, Shanghai, China; ^2^Department of Practice, Sciences, and Health Outcomes Research, University of Maryland School of Pharmacy, Baltimore, MD, United States; ^3^Division of Child and Adolescent Psychiatry, Department of Psychiatry, University of Maryland School of Medicine, Baltimore, MD, United States

**Keywords:** suicide, microsimulation, children and adolescents, mental health, public health

## Abstract

**Introduction:**

Despite considerable investment in suicide prevention since 2001, there is limited evidence for the effect of suicide prevention interventions among children and adolescents. This study aimed to estimate the potential population impact of different interventions in preventing suicide-related behaviors in children and adolescents.

**Methods:**

A microsimulation model study used data from national surveys and clinical trials to emulate the dynamic processes of developing depression and care-seeking behaviors among a US sample of children and adolescents. The simulation model examined the effect of four hypothetical suicide prevention interventions on preventing suicide and suicide attempt in children and adolescents as follows: (1) reduce untreated depression by 20, 50, and 80% through depression screening; (2) increase the proportion of acute-phase treatment completion to 90% (i.e., reduce treatment attrition); (3) suicide screening and treatment among the depressed individuals; and (4) suicide screening and treatment to 20, 50, and 80% of individuals in medical care settings. The model without any intervention simulated was the baseline. We estimated the difference in the suicide rate and risk of suicide attempts in children and adolescents between baseline and different interventions.

**Results:**

No significant reduction in the suicide rate was observed for any of the interventions. A significant decrease in the risk of suicide attempt was observed for reducing untreated depression by 80%, and for suicide screening to individuals in medical settings as follows: 20% screened: −0.68% (95% credible interval (CI): −1.05%, −0.56%), 50% screened: −1.47% (95% CI: −2.00%, −1.34%), and 80% screened: −2.14% (95% CI: −2.48%, −2.08%). Combined with 90% completion of acute-phase treatment, the risk of suicide attempt changed by −0.33% (95% CI: −0.92%, 0.04%), −0.56% (95% CI: −1.06%, −0.17%), and −0.78% (95% CI: −1.29%, −0.40%) for reducing untreated depression by 20, 50, and 80%, respectively. Combined with suicide screening and treatment among the depressed, the risk of suicide attempt changed by −0.27% (95% CI: −0.dd%, −0.16%), −0.66% (95% CI: −0.90%, −0.46%), and −0.90% (95% CI: −1.10%, −0.69%) for reducing untreated depression by 20, 50, and 80%, respectively.

**Conclusion:**

Reducing undertreatment (the untreated and dropout) of depression and suicide screening and treatment in medical care settings may be effective in preventing suicide-related behaviors in children and adolescents.

## Introduction

Despite considerable public health investment in suicide prevention since 2001,^1^ the suicide rate among children and adolescents in the United States increased significantly by over 80% from 2007 to 2017 and accounted for more than 33% of deaths in this population (1, 2). The most recent U.S. Centers for Disease Control and Prevention (CDC) vital statistics surveillance report on suicides in 2020 noted an increase among those aged between 10 and 25 whereas the suicide rate declined in older age groups (3). The rise in the number of publications on suicide prevention since 2005 (4) has largely overlooked children and adolescents, and evidence of effective interventions is quite limited compared with that in adults.

Treating mental health conditions associated with a high risk of self-harm and suicide attempt is one key strategy for reducing suicide-related behaviors (i.e., suicide attempts and suicide). Depression, one of the strongest risk factors for suicide, has efficacious treatments [e.g., antidepressants and cognitive behavior therapy (CBT)] supported by rigorous clinical trials for reducing suicidal ideation (5, 6). Given that suicidal ideation is a precursor to suicide attempts and suicide (7), it would follow that adherence to depression treatment should be effective in preventing suicide-related behaviors. However, in the U.S., 60% of children and adolescents diagnosed with depression do not receive any treatment or professional counseling services (8), and, of those who do receive some form of treatment, more than half discontinue treatment within the first 3 months when the recommended treatment duration with evidence-based therapies, including pharmacotherapy (e.g., antidepressants) and non-pharmacotherapy (e.g., cognitive behavior therapy), is 36 weeks (9, 10). Undertreatment of depression may be contributing to the increase in suicide-related behaviors among children and adolescents. Our previous findings from microsimulation modeling showed a significant association between a longer duration of antidepressant treatment and a lower risk of suicide-related behaviors (11).

Due to the rarity of events, it requires a very large sample size and longitudinal designs to study suicide. As a result, there are limited opportunities to study the effectiveness of prevention interventions in reducing suicide and suicide attempt in this population (12). To overcome the challenge, one option is to use computational methods to simulate intervention effects. Our previous study developed a microsimulation model integrating data from published clinical trials and other related data sources to investigate the association between undertreatment of depression and suicide-related behaviors in children and adolescents with depression (11) and found undertreatment of depression may be related to increased risk of suicide-related behaviors. The present study extended the microsimulation model we developed before to the general child and adolescent population in the United States to evaluate the effectiveness of minimizing the undertreatment of depression in children and adolescents in reducing suicide-related behaviors.

## Methods

A microsimulation model was developed to emulate the dynamic process of developing depression, care-seeking behaviors, and occurrence of suicidal ideation and suicide-related behaviors in a synthesized population representative of children and adolescents aged between 10 and 17 years in the United States. The study was exempt from IRB review by the University of Maryland, Baltimore Institutional Review Board.

### Data sources

Data used to parameterize the microsimulation model came from multiple sources (Supplementary Table A1). The target population of the present study was children and adolescents in the United States; therefore, the primary data sources we utilized were those that provided information for this population. Data on the sociodemographic and clinical characteristics of the target population were extracted from two nationally representative surveys, Medical Expenditure Panel Survey (MEPS, 2016–2018) and the National Comorbidity Survey–Adolescent Supplement (NCSAS 2000–2004). The efficacy of depression treatment was derived from the published results of two clinical trials which were based on adolescents in the United States: the Treatment for Adolescents with Depression Study (TADS) (5, 13, 14) and the Treatment of Resistant Depression of Adolescents (TORDIA) study (6, 15, 16). These two studies are still important references for the efficacy of depression treatment in children and adolescents in the United States. Information for suicidal ideation and suicide-related behaviors in the target population was obtained from NCSAS and CDC Fatal Injury Report (2016–2018). Information used to parameterize the microsimulation model that was not available in the above sources was obtained from the published literature that focuses on the child and adolescent population (e.g., probability of relapse of depression and probability of discontinuing treatment (see Supplementary material). Model calibration was conducted for the parameters that could not be estimated directly from the available data sources. We provide the details of model design, model parameterization, and model calibration in the Supplementary material.

### Overview of the microsimulation model

In a microsimulation model, one generates a synthetic population that reflects individuals who exhibit certain behaviors and outcomes according to a series of predefined rules. The rules are implemented on a discrete-time basis, also referred to as time steps in the model (e.g., week or month). In this study, each time step represents 1 month and our model simulated the dynamic processes of developing depression, seeking treatment for depression, and exhibiting suicidal outcomes in a synthesized population. At each time step, non-depressed individuals develop depression on a probability estimated based on their age, sex, race/ethnicity, family income, single or no-parent household, depression severity, and psychiatric comorbidities. Depressed individuals could newly initiate (i.e., no prior treatment for depression), continue, or discontinue (i.e., if having initiated treatment for depression) treatment in each time step (i.e., month). Individuals' depression symptoms, as measured by the Children Depression Rating Scale-Revised (CDRS-R score), change as reflected by the use or not of depression treatment. The details of how we calculated the change in CDRS-R scores are discussed elsewhere (11). To reflect real-world clinical comorbidities and healthcare utilization patterns, the microsimulation model permitted individuals to develop other psychiatric disorders, such as attention-deficit/hyperactivity disorder (ADHD), anxiety, conduct disorder, and alcohol/drug abuse, and to receive other medical care visits (i.e., visits for reasons other than depression). Equations used to estimate the probabilities are in Supplementary Table A2. Throughout the model process, individuals may have suicidal behaviors (i.e., suicidal ideation, suicide attempt, or suicide) or may die of non-suicide causes. The details of the development of the microsimulation model are described in the Supplementary material.

### Synthetic population

A group of individuals aged between 10 and 17 years was synthesized in the microsimulation model. Sociodemographic and family characteristics of the individuals, including age, sex, race/ethnicity, household income, single/no-parent household, parental suicide/suicide attempt, and parents' mental health conditions (i.e., depression, anxiety, alcohol, or drug abuse), were assigned to represent their distributions in the United States population. At each time step, individuals age 1 month (0.083 years) and are assigned a probability of developing depression, initiating treatment for depression (i.e., only for those with depression), discontinuing treatment for depression (i.e., only for those already on depression care) based on their age, sex, race/ethnicity, household income, psychiatric comorbidities (i.e., ADHD, anxiety, conduct disorder, and alcohol/drug abuse), single/no-parent household, parental suicide/suicide attempt, and parents' mental health conditions. At each time step, individuals can have suicidal ideation and suicide-related behaviors (i.e., suicide attempt and suicide). The probability of developing a suicidal outcome is influenced by individuals' age, sex, race/ethnicity, family income, single or no-parent household, depression severity, psychiatric comorbidities, individuals' previous suicidal ideation and suicide-related behaviors, and parental suicide-related behaviors. The details of calculating individuals' probability of suicidal ideation and suicide-related behaviors are discussed elsewhere (11).

### Model settings

We initialized 100,000 non-depressed individuals aged between 10 and 17 years. One complete simulation includes 170 months. The first 110 months was the “burn-in” period in which we obtained stabilized demographic and clinical distributions in the synthesized population. The last 60 months (5 years) contributed to the model analysis.

### Measures

#### Treatment for depression

Practice guidelines for evidence-based treatment (i.e., antidepressant, CBT, or combined therapy) of depression in children and adolescents recommend a 3-month acute-phase treatment followed by a continuation-phase treatment of at least 6 months (10, 17). In our microsimulation model, individuals who initiate depression treatment are assumed to receive antidepressants unless they were treatment-resistant, which was defined as showing no response after 3 months of treatment. The process of determining no response in a microsimulation model was discussed elsewhere (11). Treatment-resistant individuals are assumed to switch to another treatment, which could be antidepressant augmentation (i.e., add another antidepressant), CBT, or other psychotherapy. In the model, individuals can discontinue treatment in any time step (i.e., month). In the months when individuals are not receiving depression treatment, CDRS-R scores are assumed to change similar to that for individuals with untreated depression. Details of the simulation of the treatment of the depression process are discussed in our previous study (11).

For this simulation model study, we defined treated, under-treated, and untreated depression. Depressed individuals who never received any treatment were categorized as untreated depression. Receipt of at least 1 month of depression treatment defined individuals as treated. Individuals who missed at least 1 treatment month during the first 9 months of treatment, which covered acute- and continuation-phases of treatment, were categorized as under-treated.

We derived the efficacy of treatment for depression from published clinical trials and assumed that the efficacy of treatment for depression in children and adolescents observed in the clinical trials did not change over time.

#### Suicide prevention interventions

With the microsimulation model, we simulated suicide prevention interventions that aligned with recommendations of the United States Preventive Services Taskforce (USPSTF) (12), CDC (18), the US Surgeon General, and the National Action Alliance for Suicide Prevention (19). Four interventions were simulated separately: (1) screen all individuals for depression and initiate treatment in positive screens (i.e., reduce untreated depression); (2) reduce treatment dropout; (3) screen depressed individuals for suicide and initiate treatment; and (4) screen individuals (depressed and non-depressed) in medical care settings for suicide and initiate treatment. Details of each intervention are described in Table 1. Interventions 1 (i.e., screen all individuals) and 2 (i.e., reduce treatment dropout) were considered interventions to reduce the undertreatment of depression in the population.

**Table 1:** Description of the examined interventions to prevent suicide in the microsimulation model.

**Intervention description**	**Intervention simulation**	**Hypothetical intervention outcomes**
Intervention 1: Depression screening: screen children and adolescent age 10–17 in the U,S, for depression to identify depressed individuals that have never been treated and have these individuals initiate treatment for depression	Depression screening was simulated as decreased untreated depression in the synthetic population.	We hypothesized in the population, • 20% untreated depression is reduced • 50% untreated depression is reduced • 80% untreated depression is reduced
Intervention 2: Treatment adherence promotion: reduce drop out and increase completion of acute-phase treatment among children and adolescent (10–17) with depression in the U.S.	Reducing drop out of treatment for depression was simulated as increased proportion of individuals that completed the first 3 months of treatment (acute-phase treatment).	We hypothesized that the proportion of individuals who completed the first 3-month of treatment increased to 90%.^a^
Intervention 3: Suicide screening among children and adolescents with depression: screen all children and adolescents (10–17) with depression to identify and treat those at increased risk of suicide	Suicide screening among all patients with depression was simulated as decreased probability of suicide for individuals with suicidal ideation and suicide attempt who were intervened. We selected two interventions for individuals with suicidal ideation and suicide attempt based on literature: • Cognitive behavior therapy (CBT) for depressed individuals with suicidal ideation but not suicide attempt.^a^ • Brief intervention and contact (BIC) for depressed individuals with suicide attempt	We hypothesized that • Probability of suicide of depressed individuals with suicidal ideation but no suicide attempt after intervention was 0.47 times^a^ that of no intervention; • Probability of suicide of depressed individuals with suicide attempt after intervention was 0.1^b^ times that of no intervention
Intervention 4: Suicide screening in medical care settings: screen children and adolescents (10–17) in the U.S. in medical care settings (including depressed and nondepressed individuals) to identify and treat all individuals that are screened at increased risk of suicide.	The intervention was simulated in the same way with Intervention 3 except that this intervention was implemented among all individuals who attended at least one medical care visit during the 5-year model process.	We hypothesized that probability of suicide of individuals with suicidal ideation or suicide attempt after intervention was lowered in the same way as that of Intervention 3. Additionally, we hypothesized a series of different proportions of intervened individuals in medical care settings: • 20% were screened for suicide risk, all of those at increased risk of suicide were treated • 50% were screened for suicide risk, all of those at increased risk of suicide were treated • 80% were screened for suicide risk, all of those at increased risk of suicide were treated
Intervention 1 + Intervention 2	–	We hypothesized that in the population • 20% untreated depression is reduced • 50% untreated depression is reduced • 80% untreated depression is reduced And The proportion of individuals who completed the first 3 months of treatment increased to 90%.
Intervention 1 + Intervention 3	–	We hypothesized that in the population • 20% untreated depression is reduced • 50% untreated depression is reduced • 80% untreated depression is reduced And Probability of suicide after suicidal ideation or suicide attempt among the depressed individuals was lowered after intervention.
Intervention 2 + Intervention 3	–	The proportion of individuals who completed the first 12 weeks of treatment increased to 90%. And Probability of suicide after suicidal ideation or suicide attempt among the depressed individuals was lowered after intervention.

^a^With this benchmark, completion of continuation-phase treatment increased as well. ^b^ D'Anci et al. (20) and Fleischmann et al. (21).

The microsimulation model parameters (Supplementary Table A3) were adjusted to achieve the hypothetical suicide prevention intervention effects. A model without any intervention implemented was the baseline model.

#### Suicide outcomes in the population

The suicide outcomes were suicide rate and the risk of a suicide attempt. The 5-year suicide rate was calculated as the total number of suicides divided by the total number of individuals in the population using the model-estimated results from the last 60 months of the model process. The risk of suicide attempt was calculated as the number of individuals who ever attempted suicide (i.e., at least one suicide attempt) divided by the number of individuals in the synthesized population. Suicide rates and risk of suicide attempts under each scenario of the interventions were examined. The absolute change in the suicide rate and risk of suicide attempts between each of the interventions and the baseline was reported.

Suicide and suicide attempt in the synthesized population were examined for each suicide prevention intervention individually as well as for combined interventions. The reference for comparison was the baseline model, i.e., no intervention.

### Statistical analysis

Simulation for each suicide prevention intervention was repeated 20 times. The median and 95% credible intervals (CIs) were reported. To estimate the CIs, the 20 estimates were ordered, and the 2.5th and 97.5th percentiles were taken as the lower and upper bounds of the CIs. A CI that did not include 0 was considered significant.

### Sensitivity analysis

To test the robustness of the model-estimated suicidal outcomes to change in the parameters, we conducted several sensitivity analyses on the input model parameters, which were the probability of (1) developing depression, (2) initiating depression treatment, (3) dropping out of treatment, (4) developing other psychiatric disorders, and (5) having suicidal ideation. Each parameter was tested in a separate sensitivity analysis, where we adjusted the parameter by ±5 and ±20% and examined how the model-estimated suicide rate and risk of suicide attempt changed with the adjusted parameters.

## Results

### Characteristics of the synthetic population

The summary of the baseline characteristics of the synthetic population, including sources of the values, is listed in Table 2.

**Table 2:** Baseline characteristics of the synthesized population of children and adolescents aged between 10 and 17 years in the United States^a^.

**Input parameter**	**Attribute values (%)**	**Sources**
**Age**		
10 – 12	42	MEPS (2016–2018)
13 – 17	58	
**Sex**		
Female	48	MEPS (2016–2018)
Male	52	
**Race Ethnicity**		
Non-Hispanic White	49	MEPS (2016–2018)
Non-Hispanic Black	16	
Hispanic	24	
Other	11	
**Parental suicide attempt/suicide**	3	NCSAS (2000–2004)
**Single-parent household or not living with parents**	35	MEPS (2016–2018)
**Household poverty level**		
Low income (< 200% poverty line)	38	MEPS (2016–2018)
Middle income (200% – 400% poverty line)	33	
High income (> 400% poverty line)	25	
**Parental mental conditions**		
Father mental conditions	10	NCSAS (2000–2004)
Mother mental conditions	18	

### Effects of interventions

In the baseline microsimulation model, the 5-year prevalence of depression was 19.5% (95% CI: 16.6%, 26.6%), the 5-year prevalence of treated depression was 4.0% (95% CI: 3.5%, 4.4%), and the 5-year prevalence of untreated depression was 15.5% (95% CI: 13.1%, 22.2%). The 5-year suicide rate was 6.7 (95% CI: 4.8, 10.0) per 100,000, and the 5-year risk of suicide attempt was 13.9% (95% CI: 13.5%, 14.3%) (Table 3).

**Table 3:** Main 5-year estimates of the synthesized population of children and adolescents aged between 10 and 17 years in the United States from the baseline microsimulation model.

**5-year estimates**	**Model-estimated values (95% CI)**
Prevalence of depression (%)	19.5 (16.6, 22.6)
Prevalence of treated depression (%)	4.0 (3.5, 4.4)
Prevalence of untreated depression (%)	15.1 (13.1, 22.2)
Proportion of individuals completing acute-phase treatment (%)	48.8 (40.1, 56.2)
Suicide rate (per 100,000)	6.7 (4.8, 10.0)
Risk of suicide attempt (%)	13.9 (13.5, 14.3)

95% CI, 95% credible interval.

**Figure 1:**
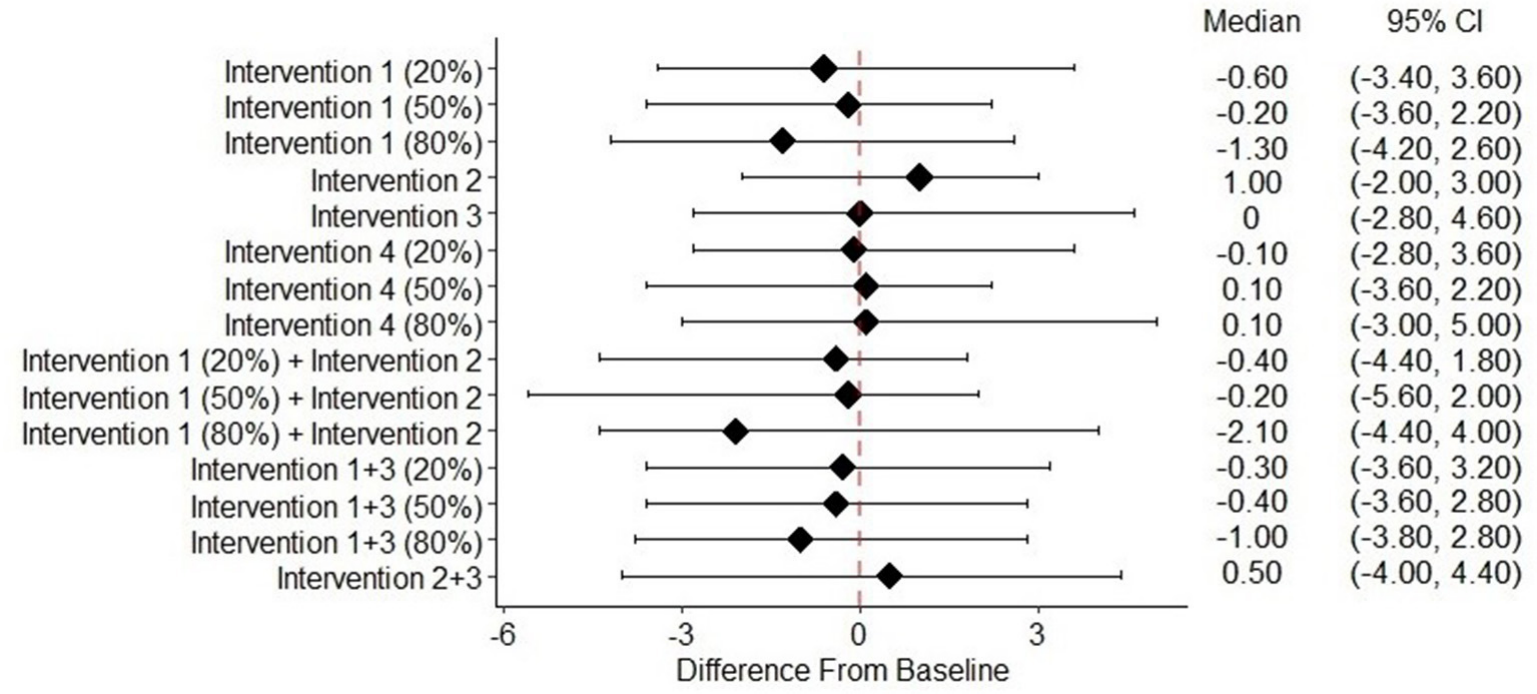
Change of suicide rate from baseline of different intervention scenarios^a^. 95% CI, 95% credible interval. ^a^Intervention (1) Depression screening. Intervention (2) Treatment adherence promotion. ^a^Intervention (3) Suicide screening among children and adolescents with depression. Intervention (4) Suicide screening among children and adolescents in medical care settings.

**Figure 2:**
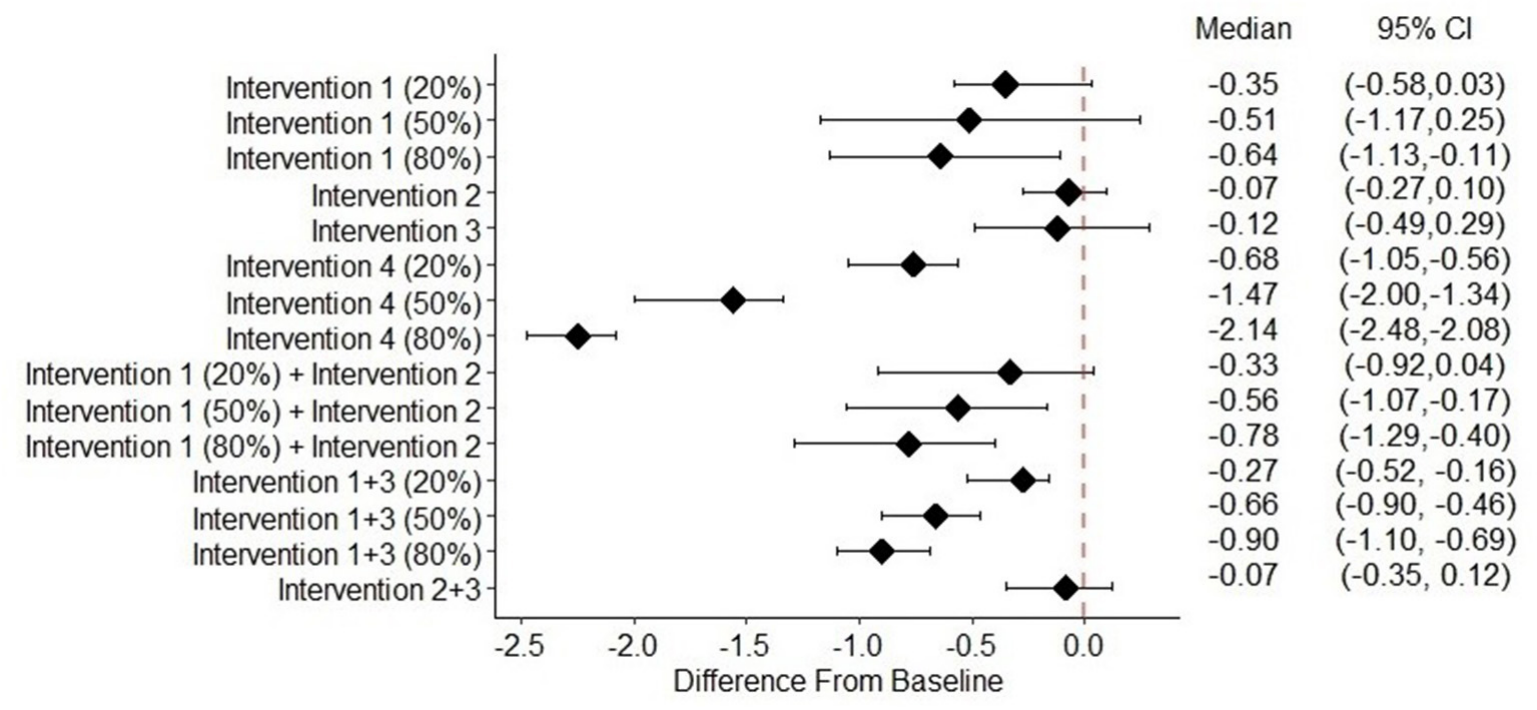
Change of risk of suicide attempt from baseline of different intervention scenarios^a^. 95% CI, 95% credible interval. ^a^Intervention (1) Depression screening. Intervention (2) Treatment adherence promotion. Intervention (3) Suicide screening among children and adolescents with depression. Intervention (4) Suicide screening among children and adolescents in medical care settings.

The suicide and risk of suicide attempts for the four suicide prevention interventions are shown in Table 4. None of the suicide prevention interventions were associated with a significant reduction in suicide rate (Figure 1). When implemented alone, screening for depression was associated with a significant reduction in the risk of suicide attempts when 80% of untreated depression in the population was reduced (−0.64%; 95% CI: −1.13%, −0.11%). Neither reducing treatment dropout nor screening and treatment for the suicide of depressed individuals showed a significant effect on reducing the risk of suicide attempts. The risk of suicide attempt significantly decreased if 20, 50, or 80% (0.68%; 95% CI: 0.56%, 1.05%, 1.47%; 95% CI: 1.34%, 2.00%, and 2.14%; 95% CI: 2.08%, 2.48%), respectively) were screened for suicide in medical settings and initiated treatment if at elevated risk of suicide (Figure 2).

**Table 4:** Model-simulated suicide rate and risk of suicide attempt in the population of each intervention.

	**Suicide rate, per 100,000 (95% CI)**	**Risk of suicide attempt, % (95% CI)**
**Depression screening**		
Reduction of untreated depression		
20%	7.10 (4.60, 10.20)	13.62 (13.43, 14.05)
50%	6.20 (4.20, 9.40)	13.48 (12.86, 13.86)
80%	5.80 (3.00, 7.40)	13.25 (12.94, 13.65)
		
Treatment adherence promotion	7.10 (6.40, 8.60)	13.83 (13.67, 13.97)
Suicide screening among children and adolescents with depression	6.70 (4.80, 9.40)	13.78 (13.66, 13.93)
Suicide screening among children and adolescents in medical care settings		
**Individuals screened**		
20%	6.80 (3.80, 8.40)	13.14 (12.98, 13.25)
50%	6.80 (5.00, 8.80)	12.31 (12.10, 12.53)
80%	6.60 (4.80, 9.80)	11.62 (11.53, 11.75)
Depression screening + Treatment adherence promotion		
**Reduction of untreated depression**		
20%	6.20 (4.20, 8.40)	13.56 (13.40, 13.67)
50%	6.10 (4.40, 8.60)	13.30 (13.11, 13.43)
80%	5.50 (3.80, 8.80)	13.09 (12.90, 13.21)
Depression screening + Suicide screening among children and adolescents with depression		
**Reduction of untreated depression**		
20%	6.40 (4.40, 9.00)	13.49 (13.29, 13.60)
50%	6.50 (4.60, 8.40)	13.09 (12.93, 13.25)
80%	6.00 (3.80, 7.60)	12.88 (12.73, 13.05)
Intervention 2 + Intervention 3	7.90 (5.00, 9.20)	13.68 (13.52, 13.90)

95% CI, 95% credible interval.

When depression screening and reducing treatment dropout were implemented together in the simulation model, we observed a greater decrease in the risk of suicide attempts compared with either intervention alone. When completion of acute-phase treatment increased to 90% (i.e., treatment dropout was reduced), the risk of suicide attempt changed by −0.33% (95% CI: −0.92%, 0.04%), −0.56% (95% CI: −1.06%, −0.17%), and −0.78% (95% CI: −1.29%, −0.40%) for reducing untreated depression by 20%, 50%, and 80%, respectively. With suicide screening and treatment among the depressed, the risk of suicide attempt changed by −0.27% (95% CI: −0.dd%, −0.16%), −0.66% (95% CI: −0.90%, −0.46%), and −0.90% (95% CI: −1.10%, −0.69%) for reducing untreated depression by 20, 50, and 80%, respectively. The combination of suicide screening and treatment for depressed individuals with reducing treatment dropout did not have a significant effect on reducing the risk of suicide attempts (Figure 2).

### Sensitivity analysis

The model-estimated suicide rate and risk of suicide attempt were generally robust to the probability of developing depression, initiating treatment for depression, dropping out of treatment, and developing other psychiatric disorders. The probability of suicidal ideation had a larger impact on the risk of suicide attempt than on the suicide rate (Table 5).

**Table 5:** Sensitivity analysis of input model parameters and the impact on model-estimated suicide rate and risk of suicide attempt^a^.

**Input parameters**	**Suicide rate**				**Risk of suicide attempt**			
	**Adjustment to the input parameters**				**Adjustment to the input parameters**			
Probability of	−20%	−5%	5%	20%	−20%	−5%	5%	20%
Suicidal ideation	8.00	7.80	7.80	8.20	12.69	13.64	14.17	14.85
	(6.20, 8.40)	(4.60, 8.4)	(5.60, 9.20)	(5.20, 9.60)	(12.59, 12.9)	(13.29, 13.70)	(14.06, 14.18)	(14.79, 14.90)
Developing depression	8.00	8.20	7.40	7.60	14.13	13.94	13.85	13.75
	(6.00, 8.80)	(6.40, 8.60)	(7.40, 9.60)	(6.00, 8.40)	(14.01, 14.26)	(13.88, 14.07)	(13.77, 14.02)	(13.64, 13.90)
Initiating treatment for depression	7.40	7.20	7.40	7.20	13.89	13.96	13.86	13.91
	(7.20, 8.80)	(5.80, 8.20)	(7.20, 9.40)	(5.60, 7.60)	(13.77, 13.98)	(13.82, 13.99)	(13.80, 13.91)	(13.78, 13.98)
Dropping out during treatment	6.40	6.40	7.60	8.20	13.88	13.90	13.91	13.94
	(6.40, 7.40)	(5.40, 8.40)	(6.20, 8.20)	(6.40, 9.00)	(13.82, 14.00)	(13.87, 14.06)	(13.81, 14.03)	(13.92, 14.00)
Developing anxiety	7.60	6.60	6.20	7.00	13.97	13.95	13.88	13.85
	(5.00, 9.80)	(6.20, 9.60)	(5.40, 8.60)	(5.80, 8.00)	(13.90, 14.00)	(13.72, 14.09)	(13.84, 13.98)	(13.79, 13.95)
Developing bipolar disorder	7.20	8.20	7.20	6.40	13.91	13.91	13.91	13.94
	(6.60, 8.60)	(5.60, 9.40)	(6.60, 9.40)	(5.60, 7.00)	(13.85, 14.10)	(13.77, 13.99)	(13.85, 14.06)	(13.83, 13.97)
Developing ADHD	9.00	7.40	8.40	7.60	13.95	13.91	13.94	13.78
	(7.80, 9.80)	(6.20, 9.40)	(6.80, 9.60)	(6.40, 8.60)	(13.85, 14.03)	(13.76, 14.13)	(13.83, 14.05)	(13.73, 13.99)
Developing conduct disorder	7.80	7.00	6.60	7.20	13.92	13.95	13.78	13.91
	(5.40, 8.80)	(6.00, 8.80)	(5.60, 7.60)	(4.60, 8.20)	(13.71, 14.01)	(13.87, 14.00)	(13.76, 13.93)	(13.86, 14.14)
Developing alcohol or drug abuse	6.80	7.40	7.80	8.00	13.91	13.89	13.90	13.87
	(5.20, 7.80)	(4.60, 8.40)	(7.00, 8.20)	(5.80, 8.60)	(13.85, 13.96)	(13.83, 13.92)	(13.85, 14.07)	(13.78, 13.92)

^a^Each sensitivity analysis was run five times, and the medians were reported.

## Discussion

Our study integrated data and scientific evidence from multiple sources using a microsimulation model to estimate the potential impact of different interventions on preventing suicide in children and adolescents. The present study is a pragmatic example of combining what is known about the efficacy of treatment (i.e., depression symptom change over time) and treatment utilization in the population (e.g., the prevalence of depressed individuals who initiate treatment; and the proportion of individuals who complete acute-phase treatment.) to estimate the risk of suicide-related behaviors in the population when no data are available to directly conduct the analysis (22). To the best of our knowledge, this is the first study that evaluates the impact of interventions that reduce undertreatment of depression on preventing suicide in children and adolescents.

A limited effect on preventing suicide-related behaviors was observed for reducing the undertreatment of depression in the population, but it should not be interpreted as the limited benefits of reducing the undertreatment of depression in children and adolescents. The results only indicate a marginal effect of treatment for depression extrapolated based on the efficacy of antidepressants. The present study assumed that the interventions achieved the desired implementation goals (i.e., reducing untreated depression or increasing the proportion of individuals that complete acute-phase treatment by a certain percentage). We did not account for healthcare providers' awareness of suicide and communication skills, patients' attitudes toward depression treatment, family's support, and access to healthcare, all of which contribute to the successful implementation of the intervention. The actual impact of reducing undertreatment in preventing suicide-related behaviors could be more variable in real-world settings.

The present study results do not fully support the CDC-recommended key suicide prevention strategy of reducing treatment attrition (18). Reducing treatment dropout alone did not significantly decrease the suicide rate or the risk of suicide attempts, but when implemented in conjunction with depression screening there was a significant reduction in the risk of suicide attempt. This suggests that the effect of reducing attrition during treatment on preventing suicide-related behaviors may be largely dependent on the treated population. In practice, reducing untreated depression (i.e., increasing the treated population) while promoting treatment continuity among those who have initiated depression treatment may be more efficient to prevent suicide attempts than implementing either one of the interventions alone.

Our finding that suicide screening and treatment in medical care settings significantly reduced the risk of suicide attempts aligns with the importance of healthcare settings as an optimal location to implement suicide prevention interventions (23). An implication of our study is that promoting access to healthcare is critical because individuals at high risk of suicide are more likely to receive treatment for suicide if they are engaged in medical care. Suicide-related behaviors are more common among children and adolescents living in areas where healthcare facilities and mental health services are limited (24–26). Facilitating access to healthcare services can be a key area to intervene in suicide prevention (18).

The present study estimated the effects of suicide screening and treatment in medical care settings assuming that an implementation goal was reached (e.g., 20% of individuals in medical care settings are screened). A more important question to answer in real-world settings is how to achieve the implementation goals. The Zero Suicide (ZS) model is a systematic approach to preventing suicide within healthcare systems, which proposes that clinicians should maximize the opportunity to identify and treat all individuals at elevated risk of suicide (27). The ZS model includes a series of system-wide strategies such as fostering a more suicide-aware environment, training staff for better care of suicide prevention, promoting patient engagement, and improving the continuity and quality of suicide care (28). The findings of our study support the importance of suicide screening (i.e., capturing individuals at risk of suicide as many as possible) emphasized by the ZS model. However, the results of the study did not directly speak to the actual impact of a systematic approach like the ZS model. Our study did not account for the influence of healthcare providers, patients' engagement in the treatment, and intervention continuity, all of which can influence the effectiveness of suicide screening and care.

The findings of the present study should be interpreted with caution. The present study aimed to provide a national-level estimate of the potential impact of various interventions in preventing suicide-related behaviors in children and adolescents. State-level estimates can be different due to some of the model parameters (e.g., probability of developing depression and probability of initiating treatment for depression) which were extrapolated from a national survey conducted 15 years ago (i.e., NCSAS), which may not reflect the most up-to-date estimates for these parameters. We used data from NCSAS to derive an association between risk factors and suicide-related behaviors, and we assumed that such a relationship would mostly remain stable over time. Moreover, the sensitivity analysis suggested that our model-estimated suicide rate and risk of suicide attempt were robust to these parameters. Important social determinants, such as healthcare access, medication beliefs, social stigma (e.g., peer pressure), and community support factors associated with mental health service utilization and suicide-related behaviors (29, 30), were not accounted for in the simulation model because there were no data available. We assumed that the intervention effect, once implemented, would last until the end of the study, which may not be the case. In real-world settings, successful implementation of the intervention may rely on effective communication and ongoing engagement with patients. Finally, the microsimulation model assumes a constant probability of developing depression, other psychiatric disorders, and suicide-related behaviors across different times of the year. In real-world settings, the onset of mental health issues and suicide-related behaviors may display seasonal variability, e.g., schools and holidays (31).

## Conclusion

Combined interventions that reduce the undertreatment of depression, including reducing untreated depression and attrition from treatment, in the population may be more effective than implementing either intervention alone. Suicide screening and intervention in medical care settings may be effective in reducing suicide-related behaviors in children and adolescents.

## Data availability statement

The original contributions presented in the study are included in the article/Supplementary material, further inquiries can be directed to the corresponding author.

## Author contributions

CZ: conceptualization, methodology, data curation, formal analysis, and writing—original draft preparation. ZZ: methodology, formal analysis, and writing—reviewing and editing. JS, WC, and GR: methodology and writing—reviewing and editing. SdR: supervision, methodology, and writing—reviewing and editing. All authors contributed to the article and approved the submitted version.
