# Supplementary material for: Impact of different interventions on preventing suicide and suicide attempt among children and adolescents in the United States: a microsimulation model study
Source: Front Psychiatry. 2023 Jun 2;14:1127852. doi: 10.3389/fpsyt.2023.1127852 (PMC10275605; doi:10.3389/fpsyt.2023.1127852)

# **Development of a Microsimulation Model that Simulates the Dynamics of Depression Care Seeking Behaviors in Children and Adolescents**

# **Model Overview**

A microsimulation model to simulate the development of depression and dynamic care-seeking behaviors of children and adolescents was constructed where each time step of the model represented one month. In a simulation run, individuals with depression will initiate, continue or discontinue treatment for depression during the course of depression. The model parameters were derived from the following key data sources: MEPS (2016-2018), NCSAS (2000-2004), published landmark clinical trials (i.e., TADS and TORDIA studies), United States Census data (2016) and the CDC Fatal Injury Report (2016-2018). For parameters that could not be derived from the listed data sources, we extrapolated them from published literature.

## **Attributes and Behaviors of Individuals**

A group of individuals age 10 -17 years old. Sociodemographic and family characteristics, including age, sex, race/ethnicity, household income, single/no-parent household, parental suicide/suicide attempt, and parents’ mental health conditions (i.e., depression, anxiety, alcohol or drug abuse), were assigned to represent their distributions in the United States population. In each time step, individuals age one month (0.083 years), and are assigned a probability of developing psychiatric disorders (i.e., depression, bipolar disorder, anxiety, ADHD, conduct disorder and alcohol/drug abuse), initiating treatment for depression (i.e., only for those with depression), discontinuing treatment for depression (i.e., only for those already on depression care) and having medical care visits (i.e., outpatient, emergency and inpatient settings) in addition to treatment for depression. In each time step, individuals may have suicidal behaviors (i.e., suicidal ideation, suicide attempt or suicide) or may die of non-suicide causes.

## **Model Process**

In each time-step (one month), the following sub-models are executed to update the characteristics of individuals:

1. Developing depression. In this sub-model, nondepressed individuals have a probability of developing depression and being characterized as depressed individuals. Among depressed individuals, the following steps are applied:
2. Initiating treatment for depression. In this sub-model, depressed individuals not on treatment for depression have a probability of initiating treatment for depression. This sub-model is not executed for non-depressed individuals.
3. Dropping out of treatment for depression. If an individual is already on treatment for depression, he/she has a probability of dropping out the current treatment session. This sub-model is not executed for non-depressed individuals.
4. Changing depressive symptoms. In this sub-model, CDRS-R scores of depressed individuals change (i.e., worsened or improved). This sub-model is not executed for non-depressed individuals.
5. Achieving remission or recovery. Depressed individuals may remit or recover from depression according to the following rules:
   1. If the CDRS-R score of an individual falls below 28, the individual is considered in remission.
   2. If an individual has maintained remission for two consecutive months, the individual recovers and becomes a nondepressed individual.

This sub-model is not executed for non-depressed individuals.

1. Developing other psychiatric disorders. Both non-depressed and depressed individuals have a probability of developing other non-depression psychiatric disorders. These psychiatric disorders are considered chronic. Therefore, once individuals develop one of these psychiatric disorders, the disorder(s) will persist until leaving the cohort.
2. Having medical care visits. Both non-depressed and depressed individuals have a probability of seeking medical care in addition to treatment for depression.
3. Having suicidal ideation. Both non-depressed and depressed individuals have a probability of experiencing suicidal ideation.
4. Having suicide attempt. Both non-depressed and depressed individuals have a probability of attempting suicide.
5. Suicide. Both non-depressed and depressed individuals have a probability of suicide.
6. Dying of other causes. Both non-depressed and depressed individuals have a probability of experiencing a non-suicide-related death.
7. Removing individuals who die or reach age 18 (in non-depressed and depressed individuals).
8. Re-creation of a nondepressed individual for each of the individuals removed.

# **Model Parameterization**

Equations to calculate the probabilities for each of the sub-models above were determined using the best available data sources.

## **Developing Depression**

The following equations were used to estimate the probability of developing depression (i.e., the probability that nondepressed individuals develop depression in a time step):

$logit\left( \mu_{\left( depression=1, 1 year \right)} \right)=\beta_{d0}+\beta_{d1}age+\beta_{d2}male+\beta_{d3}nhw+\beta_{d4}nhb+ \beta_{d5}hisp+\beta_{d6}lowIncome+\beta_{d7}midIncome+\beta_{d8}singleParent+\beta_{d9}fathermental+\beta_{d10}mothermental+\beta_{d11}bipolar+\beta_{d12}ADHD+\beta_{d13}cdodd+\beta_{d14}anxiety+\beta_{d15}adAbuse$ (1)

$\mu_{\left( depression=1, 1 year \right)}=\frac{e^{logit\left( \mu_{\left( depression=1, 1 year \right)} \right)}}{e^{logit\left( \mu_{\left( depression=1, 1 year \right)} \right)}+1}$ (2)

$P_{(depression=1, one time step)}= 1-e^{\left( -\frac{\mu_{\left( depression=1, 1 year \right)}}{12} \right)}$ (3)

In Equation (1),

*age* is a continuous variable of the individuals’ age in the current time-step

*Sex:*

*male* is binary male = 1, female = 0

*Race/Ethnicity:*

*hisp* for Hispanic is binary: hisp = 1, nhb = 0, nhw = 0

*nhb* for non-Hispanic Black is binary: hisp = 0, nhb = 1, nhw = 0

*nhw* for non-Hispanic White is binary: hisp = 0, nhb = 0, nhw = 1

Other race/ethnicity: hisp = 0, nhb = 0, nhw = 0)

*Household Income:* two dummy variables were created to represent the three-level (i.e., low, middle and high) household income:

*lowIncome* is binary: lowIncome = 1, midIncome = 0

*middle income* is binary: lowIncome = 0, midIncome = 1

high income is represented as lowIncome = 0, midIncome = 0

*Single Parent Househol*d:

*singleParent* = 1 if the individual is in a single/no parent home, else *singleParent* = 0.

*Parent Mental Health Conditions ever:*

*fathermenta*l = 1 if the individual’s father has any mental conditions, else *fathermental* = 0.

*mothermental* = 1 if the individual’s mother has any mental conditions, else *mothermental* = 0.

*Agent Mental Health Conditions:*

*bipolar* = 1 if the individual has bipolar disorder, else *biplar* = 0.

*Adhd* = 1 if the individual has ADHD, else *adhd* = 0.

*Cdodd* = 1 if the individual has conduct and/or oppositional defiant disorder, else *cdodd* = 0.

*Anxiety* = 1 if the individual has anxiety disorder, else *anxiety* = 0.

*adAbuse* = 1 if the individual has alcohol or drug abuse, else *adAbuse* = 0.

In Equation (1), logit( $\mu_{\left( depression=1, 1 year \right)}$) is an estimate of the likelihood of depression over 12 months. Equation (2) calculates the depression rate during 12 months, assuming 12-month follow-up for all subjects in the sample. The probability of developing depression at one time step was thus calculated with Equation (3), where $\mu_{\left( depression=1, 1 year \right)}/12$ converts the yearly rate of depression to a monthly rate.

Equation (1) was estimated using the NCSAS data. The rationale for choosing NCSAS over other possible data sources, such as MEPS, is that the NCSAS includes both treated and untreated (i.e., never received any treatment) depressed individuals, which provides more comprehensive information from which to estimate the probability of developing depression than MEPS, which only provides an estimate of those who had some medical visits or prescriptions related to the reported condition. The MEPS questions to capture depression status (i.e., Patient Health Questionnaire-2(PHQ-2)) are not administered to children, only adults. Therefore, depression status for untreated is not available in the MEPS data.

## **Treatment for depression**

*Initiating Treatment for Depression*

Depressed individuals who are not on treatment for depression have a probability of initiating treatment for depression. The probability of initiating treatment for depression was calculated using the following equations:

$logit\left( \mu_{\left( treatment=1, 1 year \right)} \right)=\beta_{t0}+\beta_{t1}age+\beta_{t2}male+\beta_{t3}nhw+\beta_{t4}nhb+ \beta_{t5}hisp+\beta_{t6}lowIncome+\beta_{t7}midIncome+\beta_{t8}singleParent+\beta_{t9}fathermental+\beta_{t10}mothermental+\beta_{d11}mental$ (4)

$\mu_{\left( treatment=1, 1 year \right)}=\frac{e^{logit\left( \mu_{\left( treatment=1, 1 year \right)} \right)}}{e^{logit\left( \mu_{\left( treatment=1, 1 year \right)} \right)}+1}$ (5)

$P_{(treatment=1, one time step)}= 1-e^{\left( -\frac{\mu_{\left( treatment=1, 1 year \right)}}{12} \right)}$ (6)

In this study, the probability of initiating treatment for depression after developing depression was approximated as the probability of initiating treatment for a mental condition after developing the mental conditions. This is based on the assumption that an individual can initiate treatment for depression when seeking care for other psychiatric disorders. Equation (4) estimates the logit of initiating treatment for depression. In Equation (4), “mental” is a composite variable to indicate if an individual has any other mental health conditions (i.e., depression, bipolar disorder, anxiety, ADHD, conduct disorder and alcohol/drug abuse).

Equation (4) was estimated using the NCSAS data. The NCSAS data include individuals who were treated and never treated, which enables one to estimate the probability for one to initiate treatment when developing mental conditions (i.e., the probability that an untreated individual initiates treatment). A depressed individual was defined as treated depression once he/she initiates treatment for depression for the first time. Depressed individuals that never initiated treatment for depression were defined as untreated.

*Dropping Out of a Treatment Month*

Depressed individuals receiving treatment for depression have a probability of dropping out of the current treatment month. The probability of dropping a treatment month was calculated based on the following equations:

$logit\left( \mu_{\left( drop=1, 1 year \right)} \right)=\beta_{drop0}+\beta_{drop1}adAbuse+\beta_{drop2}ADHD+\beta_{drop3}severeDepression$ (7)

$\mu_{\left( drop=1, 1 year \right)}=\frac{e^{logit\left( \mu_{\left( drop=1, 1 year \right)} \right)}}{e^{logit\left( \mu_{\left( drop=1, 1 year \right)} \right)}+1}$ (8)

$P_{(drop=1, one time step)}= 1-e^{\left( -\frac{\mu_{\left( drop=1, 1 year \right)}}{12} \right)}$ (9)

In Equation (7), “severeDepression” represents severity of depressive symptoms (severe depression = 1, mild to moderate depression = 0).

Since there is no available data source to directly estimate the association between treatment discontinuation and an individual’s characteristics, βs in Equation (7) were derived from a published meta-analysis study that evaluated factors associated with medication adherence among children and adolescents with mental health conditions.^1^ The study found significant impact of alcohol/drug abuse, ADHD and depression severity on treatment nonadherence. The coefficients in Equation (7) were extrapolated by converting (i.e., taking natural log of) the reported ORs in the study into the coefficients used in the equation. The intercept in Equation (7) was assumed 0 at the beginning and determined in the calibration process. The final equations used in the microsimulation model are listed in Appendix Table 2.

## **Depressive Symptoms Change**

CDRS-R scores measure the severity of depressive symptoms. Depressive symptom severity was categorized based on established CDRS-R score cut-off points used in published clinical trials,^2,3^ where 35-60 defined mild to moderate depression and above 60 defined severe depression.

CDRS-R scores were assumed to change as a linear function of natural log of days since depression onset. The following equations were applied to update the CDRS-R score at each time step:

${CDRS-R Score}_{i1}= {Baseline CDRS-R Score}_{i}+\beta_{s_{ij}}\ln\left( j*30 \right)+\varepsilon_{ij}$ where j = 1 (10)

${CDRS-R Score}_{ij}= {CDRS-R Score}_{i j-1}+\beta_{s_{ij}}\ln\left( \frac{j}{j-1} \right)+\varepsilon_{ij}$ where j ≥ 2 (11)

In Equations (10) and (11), i and j represent an individual i at the j^th^ month since depression onset. Equation (10) was used to calculate the CDRS-R score change from baseline to the end of the first month after depression onset. Equation (11) was used to update the CDRS-R scores since the second month after depression onset. $\beta_{s_{ij}}$ is the change of the CDRS-R score with every month. The βs correspond to the treatment for depression status (i.e., on treatment or not) in the current time step. The random error, $\varepsilon_{ij},$ follows a normal distribution N (0, 0.1). Variability in individual trajectories of depressive symptom change was accounted for by assuming βs among depressed individuals were distributed with a certain mean (i.e., fixed effect) and variance (i.e., random effect).

The process for determining the mean and variance of βs was described in our previous work.^4^ The approach to simulate relapse of depression was described in our previous work.^4^

## **Other Psychiatric Disorders**

Nondepressed and depressed individuals can develop other psychiatric disorders, and the probabilities of developing a psychiatric disorder (excluding depression) were calculated with the following equations:

$logit\left( \mu_{\left( {comorbid}_{k}=1, 1 year, depression status \right)} \right)=\beta_{c_{k}h0}+\beta_{c_{k}h1}child+\beta_{c_{k}h2}male+\beta_{c_{k}h3}nhw+\beta_{c_{k}h4}nhb+ \beta_{c_{k}h5}hisp+\beta_{c_{k}h6}lowIncome+\beta_{c_{k}h7}midIncome+\beta_{c_{k}h8}singleParent+\beta_{c_{k}h9}fathermental+\beta_{c_{k}h10}mothermental$ (12)

$\mu_{\left( {comorbid}_{k}=1, 1 year, by depression status \right)}=\frac{e^{logit\left( \mu_{\left( {comorbid}_{k}=1 over12 months, depression status \right)} \right)}}{e^{logit\left( \mu_{\left( {comorbid}_{k}=1 over12 months, depression status \right)} \right)}+1}$ (13)

$P_{({comorbid}_{k}=1, one time step)}= 1-e^{\left( -\frac{\mu_{\left( {comorbid}_{k}=1, 1 year, depression status \right)}}{12} \right)}$ (14)

Where, *k* represents each psychiatric disorder (1=bipolar disorder, 2=ADHD, 3=CD/ODD, 4=anxiety, 5=alcohol/drug abuse), and *h* represents depression status (1=depressed, 0=non-depressed). A non-depressed individual that developed bipolar disorder was characterized as a depressed individual.

Coefficients in Equations (12) for bipolar disorders, ADHD, conduct disorders, and anxiety disorders were estimated directly from MEPS 2016-2018, respectively, to obtain the most up-to-date information. Since the MEPS data do not report alcohol/drug abuse, we referred to NCSAS to estimate the coefficients for this disorder. The parameterized equations used in the microsimulation model are listed in Appendix Table 2.

## **Medical Care Visits**

Medical care visits include any outpatient (including hospital outpatient department, clinics and other office-based visits), emergency department and inpatient visits individuals have in addition to treatment for depression. The purpose of including this sub-model is to estimate medical visits in the population. All individuals, including depressed and non-depressed, have a probability of medical care visits in a time step. The probability of having a medical care visit(s) was calculated using the following equations:

$log\left( \frac{\mu_{\left( {Visit}_{kh}=1, depression status \right)}}{1 year} \right)=\beta_{v_{k}h0}+\beta_{v_{k}h1}age+\beta_{v_{k}h2}male+\beta_{v_{k}h3}nhw+\beta_{v_{k}h4}nhb+ \beta_{v_{k}h5}hisp+\beta_{v_{k}h6}lowIncome+\beta_{v_{k}h7}midIncome$ (15)

$\mu_{\left( {Visit}_{kh}=1, 1 year \right)}= e^{log\left( \frac{\mu_{\left( {Visit}_{kh}=1,depression status \right)}}{1 year} \right)}$ (16)

$P_{({Visit}_{k}=1, one time step)}= 1-e^{\left( -\frac{\mu_{\left( {Visit}_{kh}=1,depression status, 1 year \right)}}{12} \right)}$ (17)

Equation (15) is a Poisson regression model that was used to estimate the rate of medical care visits in a year $\mu_{\left( {Visit}_{kh}=1 \right)}$, and Equation (17) estimates the probability of having a medical care visit in a time step.

In the equations above, k represents the type of medical care visits (1=outpatient, 2=emergency, 3=inpatient), and h represents depression status (1 = treated depression, 0 = untreated depression or nondepressed). Coefficients for Equation (15) were directly estimated based on the MEPS data. The parameterized equations used in the ABM are listed in Appendix Section 8.5.

## **Suicidal Events**

Suicidal events include suicidal ideation, suicidal attempt and suicide. Different approaches were applied to estimate the probability of suicidal ideation, suicide attempt, and suicide, to account for the difference among these suicidal behaviors.

The equation used to calculate the probability of having suicide ideation was discussed earlier in our previous work.^4^

In the microsimulation model, the probabilities of suicide attempt and suicide were re-calculated at each time step based on suicidal ideation status (i.e., having suicidal ideation or not) and depression status. The process for determining the probability of suicide attempt and suicide was described in our previous work.^4^ Probabilities of suicide attempt and suicide are listed in Appendix Table 2.

## **Death and Re-creation of Individuals**

The synthetic population is an open population where individuals leave because of death or age (18 years old) and new individuals enter.

In addition to death caused by suicide, individuals may die of non-suicidal causes in each time step. Probability of dying at one time step was extrapolated from the United States census data (Appendix Table 2).

To maintain a constant number of individuals in the synthetic population, a new individual aged 10 years old is re-created (i.e., enters the cohort) with every individual that died or leaves the cohort at age 18 years old.

# **Model Calibration**

Calibration was conducted for the parameters that cannot be estimated directly from available data source.

There was no data to directly estimate the probability of discontinuing treatment for depression among the depressed individuals. Therefore, coefficients in Equation (7), based on which the probability of discontinuing treatment for depression was calculated, were extrapolated from published literature assuming an intercept of 0 before calibration. The intercept of Equation (7) was then adjusted until the 95% CI (calculated based on 20 simulation runs) of the model-estimated proportion of individuals that completed the first 12 weeks of treatment (i.e., acute-phase treatment) included the empirically observed value.^5^

Although studies have shown that severe depression is more likely associated with risk of suicidal behaviors,^6^ no information is available to quantify the association between depression severity and the probability of suicide. We assumed that there was a certain cut-off point in the CDRS-R score, above which resulted in increased probability of suicide and suicide attempt for the depressed individuals compared with that in the non-depressed individuals. Therefore, different cut-off points from 60 to 90 were tested until the 95% credible interval (calculated based on 20 simulation runs) of the model-estimated suicide rate included the suicide rate obtained from CDC Fatal Injury Report (2016-2018). The final cut-off point to use in the model was 80.

**Appendix Table 1 Model Parameters, Value Assignment Rules, Update Rules and Data Sources for Parameterization of the Microsimulation Model**

| Parameter | Values | Attribute assignment rules | Update Rules | Data sources |
| --- | --- | --- | --- | --- |
| Characteristics of Individuals | | | | |
| Age | 10-18 (in months) | Input at initialization. | Age increased by one month at each time step. | MEPS 2016-2018 |
| Sex | Male; Female | Input at initialization. | Remain unchanged. | MEPS 2016-2018 |
| Race/ethnicity | Non-Hispanic White;  Non-Hispanic Black;  Hispanic;  Other. | Input at initialization. | Remain unchanged. | MEPS 2016-2018 |
| Household income | Low income (< 200% poverty line) | Input at initialization. | Remain unchanged. | MEPS 2016-2018 |
|  | Middle income (200% - 400% poverty line) | Input at initialization. | Remain unchanged. | MEPS 2016-2018 |
|  | High income (> 400% poverty line) | Input at initialization. | Remain unchanged. | MEPS 2016-2018 |
| Single or no parents household | Yes/No | Input at initialization. | Remain unchanged. | MEPS 2016 - 2018 |
| Parental suicide or suicide attempt | Yes/No | Input at initialization. | Remain unchanged. | NCSAS 2000-2004 |
| Parental mental conditions |  |  |  |  |
| Father mental conditions | Yes/No | Input at initialization. | Remain unchanged. | NCSAS 2000-2004 |
| Probability of dying | 0-1 | Assigned based on age (under 15 years old and 15 years or older) | Re-assigned at each time step. | US National Census 2016 |
| Probability of mental illness |  |  |  |  |
| Mother mental conditions | Yes/No | Input at initialization. | Remain unchanged. | NCSAS 2000-2004 |

**Appendix Table 1 continued**

| Depression | 0-1 | Calculate based on age, sex, race/ethnicity, household income, single or no parental household, father’s mental conditions, mother’s mental conditions, status of bipolar disorder, anxiety, ADHD, conduct disorder, and alcohol or drug abuse. | Re-calculated at each time step. | MEPS 2016-2018 |
| --- | --- | --- | --- | --- |
| Bipolar disorder | 0-1 | Calculate based on age, sex, race/ethnicity, household income, single or no parental household, father’s mental conditions, and mother’s mental conditions, by depression status. | Re-calculated at each time step. | MEPS 2016-2018 |
| Anxiety | 0-1 |  | Re-calculated at each time step. | MEPS 2016-2018 |
| ADHD | 0-1 |  | Re-calculated at each time step | MEPS 2016-2018 |
| CD/ODD | 0-1 |  | Re-calculated at each time step. | MEPS 2016-2018 |
| Alcohol or drug abuse | 0-1 |  | Re-calculated at each time step. | MEPS 2016-2018 |
| Probability of continuing the depression treatment | 0-1 | Calculated based on alcohol or drug abuse, ADHD and severity of depression. | Re-calculated at each time step after agents had initiated depression treatment. | Timlin U et al. 2015^1^ |

**Appendix Table 1 continued**

| CDRS-R Score (baseline) | 35-113 | Baseline CDRS-R score for agents that developed depression was assigned based on the estimated distribution of CDRS-R scores in depressed population. | Assigned when agents developed depression. | Appendix Section 8.1.2.2 |
| --- | --- | --- | --- | --- |
| CDRS-R score monthly change | See Appendix Section 8.1.2.2 | CDRS-R score changed as a function of the natural log of days since depression by treatment status: 1) on treatment; 2) off treatment. | Re-assigned at each time step based on agents’ treatment status. | Appendix Section 8.1.2.2 |
| Probability of outpatient visits | 0-1 | Calculated based on age, sex, race/ethnicity, household income by depression status. | Re-calculated at each time step. |  |
| Probability of emergency visits | 0-1 |  | Re-calculated at each time step. |  |
| Probability of hospitalization | 0-1 |  | Re-calculated at each time step. |  |
| Probability of suicidal ideation | 0-1 | Calculated based on age, sex, race/ethnicity, household income, single or no parents household, parental suicide or suicide attempt, severity of depression, psychiatric comorbidities (ADHD, bipolar disorder, alcohol and drug abuse, CD/ODD). | Re-calculated at each time step. |  |

**Appendix Table 1 continued**

| Probability of attempting suicide | 0-1 | Assigned based on depression status and suicidal ideation status. | Re-assigned at each time step. | Appendix Section 8.1.2.3 |
| --- | --- | --- | --- | --- |
| Probability of completed suicide | 0-1 | Assigned based on age, sex, depression status, suicidal ideation status and previous suicide attempt. | Re-assigned at each time step. | Appendix Section 8.1.2.3 |

**Appendix Table 2 Values of Input Parameters and Parameterized Equations Applied in the Model**

| **Model Parameters** | **Values** |
| --- | --- |
| Age | 10-12: 42%  13-17: 58% |
| Sex | Male: 52%  Female: 48% |
| Race/ethnicity | Non-Hispanic White: 49% Non-Hispanic Black: 16%  Hispanic: 24%  Other: 11% |
| Household income |  |
| Low income (< 200% poverty line) | 38% |
| Middle income (200% - 400% poverty line) | 33% |
| High income (> 400% poverty line) | 25% |
| Single or no parents household | 35% |
| Parental suicide or suicide attempt | 3% |
| Parental mental conditions |  |
| Father mental conditions | 10% |
| Mother mental conditions | 18% |
| Probability of dying (non-suicidal) |  |
| <15 years old | 1.3/100000 (1 month) |
| ≥15 years old | 6.6/100000 (1 month) |
| Equation (1) | Logit (µ _(depression=1, 1 year)_) = -3.689 + 0.085*age +(-0.104)*male +(-0.334)*nhw + 0.018*nhb +(-0.035)*hisp + (-0.146)*lowIncome + (-0.134)*midIncome + (-0.798)*singleParent +0.608*fathermental + 1.088*mothermental +0.852*bipolar +0.525*ADHD +1.075*cdodd + 2.017*anxiety +0.436*adAbuse |
| Equation (4) | Logit(µ _(treatment = 1, 1 year)_) = -2.40 + 0.006*age + male*(0.2562) + hisp*(-1.048) + nhb*(-0.8702) + nhw*(0.3692) + lowIncome*(-0.2275) + midIncome*(0.0752) + singleParent*(0.5599) + fathermental*(-0.0118) + mothermental*0.312 + mental*(-0.0776) |
| Equation (7) | Logit (µ _(drop-out=1, 1 year)_) = 1.2 + (-0.198)*adAbuse + (-0.4943)*adhd + (-0.4005)*severeDepress |
| Equation (12) |  |

**Appendix Table 2 continued**

| Among the depressed | Logit (µ _(bipolar=1, 1 year)_) = -4.39 + 0.4325*child + 0.7671*male + 1.4777*hisp + (-14.336)*nhb + 0.5204*nhw + (-0.3227)*lowIncome + (-2.1362)*midIncome + 0.5206*singleParent + 0.0709*fathermental+ 0.6065*mothermental |
| --- | --- |
|  | Logit (µ _(ADHD=1, 1 year)_) = -3.11 + 0.1851*child + 0.5866*male + 1.1620*hisp+ 0.7555*nhb + 2.2763*nhw + 0.6750*lowIncome + 0.0966*midIncome + (-0.6773)*singleParent + (-0.6952)*fathermental + 0.7656*mothermental |
|  | Logit (µ (conduct disorder=1, 1 year)) = -5.28 + (-0.0677)*child + (-1.3373)*male + (-1.0993)*hisp + (-0.7986)*nhb + *(0.6521)*nhw  +(-1.2049)*lowIncome + (-0.8100)*midIncome + 2.4388*singleParent + 1.2940*fathermental + 2.1972*mothermental |
|  | Logit (µ _(anxiety=1, 1 year)_) = -0.081 + (-0.0957)*child + (-0.6614)*male + (-0.2516)*hisp + (-1.9338)*nhb + 0.4441*nhw + (-0.8071)*lowIncome + (-0.1133)*midIncome + 0.7325*singleParent + 0.2095*fathermental + (-0.5628)*mothermental |
|  | Logit (µ _(alcohol/drug abuse=1, 1 year)_) = -2.079 + (-0.011)* child +0.214*male +(0.869)*nhw + (-0.293)*nhb + 1.257*hisp + (-0.257)*lowIncome + (-0.053)*midIncome + (-0.357)*singleParent +0.706*fathermental + 0.056*mothermental |
| Among the nondepressed | Logit (µ _(bipolar=1, 1 year)_) = -4.39 + 0.9834*child + 0.2828* male + (-1.5008)*hisp + (-2.6462)* nhb + (-0.5630)* nhw + 1.7849* lowIncome + 1.9161*midIncome + 0.7996* singleParent + (-0.1837)* fathermental + 1.0058* mothermental |
|  | Logit (µ _(ADHD=1, 1 year)_) = -3.105 + (-0.2982)*child + 0.9418*male + 0.00789*hisp + 0.4007*nhb + 0.8440*nhw + 0.2130*lowIncome + (-0.0778)*midIncome + 0.3539*singleParent + 0.3941*fathermental + 0.7795*mothermental |
|  | Logit (µ _(conduct disorder=1, 1 year)_) = -5.276 + (-0.3431)*child + 0.8489*male+ 0.3793*hisp + 0.1098*nhb + 0.7448*nhw + 0.5535*lowIncome + (-0.3037)*midIncome + 0.2092*singleParent + 0.4282*fathermental + 1.1450*mothermental |

**Appendix Table 2 continued**

|  | Logit (µ _(anxiety=1, 1 year)_) = -0.081 + 0.1696*child + (-0.3188)*male + 0.0631*hisp + (-0.4254)*nhb + 1.4326*nhw + 0.00042*lowIncome + (-0.1611)*midIncome + 0.0498*singleParent + 0.9132*fathermental + 1.1522*mothermental |
| --- | --- |
|  | Logit (µ _(alcohol/drug abuse=1, 1 year)_) = -2.079 + 0.5481*child + 0.4541*male + 0.5213*hisp +(-0.6268)*nhb + 1.0474*nhw + (-0.3791)*lowIncome + (-0.2804)*midIncome + 0.2775*singleParent + 1.0327*fathermental + 0.749*mothermental |
| Equation (15) |  |
| Outpatient visits |  |
| Among the depressed | Log (µ _(outpatient = 1)_/1 year) = 0.339 + (-0.0709)*age + (0.7209)*male + (-0.7374)*nhw + (-0.6024)*nhb + (-0.1397)*hisp + (-0.7956)*lowIncome + (-1.1663)*midIncome |
| Among the nondepressed | Log (µ _(outpatient = 1)_/1 year) = -3.186 + (0.0353)*age + (0.0991)*male + (1.1040)*nhw + (0.1211)*nhb + (0.4544)*hisp + (-0.0019)*lowIncome + (-0.0862)*midIncome |
| Emergency visits |  |
| Among the depressed | Log (µ _(emergency = 1)_/1 year) = 0.150 + (-0.1571)*age + (0.7894)*male + (-0.3319)*nhw + (-0.4554)*nhb + (0.0925)*hisp + (-0.0828)*lowIncome + (-0.5221)*midIncome |
| Among the nondepressed | Log (µ _(emergency = 1)_/1 year) = -2.934 + (0.0513)*age + (-0.0185)*male + (-0.1201)*nhw + (-0.1057)*nhb + (-0.3181)*hisp + (0.4870)*lowIncome + (0.2492)*midIncome |
| Inpatient visits |  |
| Among the depressed | Log (µ _(inpatient= 1)_/1 year) = -2.277 + (-0.0861)*age + (1.1673)*male + (-0.2681)*nhw + (-0.0835)*nhb + (-0.3972)*hisp + (0.0442)*lowIncome + (-0.6281)*midIncome |
| Among the nondepressed | Log (µ _(inpatient= 1)_/1 year) = -6.127 + (0.0944)*age + (0.0790)*male + (0.3923)*nhw + (0.0468)*nhb + (0.3828)*hisp + (0.4247)*lowIncome + (0.1272)*midIncome |
| Probability of Suicide Attempt (depressed)^4^ |  |
| With suicidal ideation | 0.0216 |

**Appendix Table 2 continued**

| Without suicidal ideation | 0.0084 |
| --- | --- |
| Probability of Suicide Attempt (nondepressed)^4^ |  |
| With suicidal ideation | 0.0056 |
| Without suicidal ideation | 0 |
| Probability of Suicide (depressed)^4^ |  |
| Female, without suicidal ideation or suicide attempt | Age < 13: 1.11/100000 (1 month)  Age>=13: 3.90/100000 (1 month) |
| Female, with suicidal ideation but no suicide attempt | Age < 13: 2.60/100000 (1 month)  Age>=13: 11.66/100000 (1 month) |
| Female, with suicide attempt | Age < 13: 4.42/100000 (1 month)  Age>=13: 15.52/100000 (1 month) |
| Male, without suicidal ideation or suicide attempt | Age < 13: 3.53/100000 (1 month)  Age>=13: 12.95/100000 (1 month) |
| Male, with suicidal ideation but no suicide attempt | Age < 13: 8.28/100000 (1 month)  Age>=13: 38.36/100000 (1 month) |
| Male, with suicide attempt | Age < 13: 14.05/100000 (1 month)  Age>=13: 50.81/100000 (1 month) |
| Probability of Suicide (nondepressed)^4^ |  |
| Female, without suicidal ideation or suicide attempt | Age < 13: 0.02/100000 (1 month)  Age >= 13: 0.16/100000 (1 month) |
| Female, with suicidal ideation but no suicide attempt | Age < 13: 0.06/100000 (1 month)  Age >= 13: 0.47/100000 (1 month) |
| Female, with suicide attempt | Age < 13: 0.09/100000 (1 month)  Age >= 13: 0.63/100000 (1 month) |
| Male, without suicidal ideation or suicide attempt | Age < 13: 0.04/100000 (1 month)  Age >= 13: 0.31/100000 (1 month) |
| Male, with suicidal ideation but no suicide attempt | Age < 13: 0.12/100000 (1 month)  Age >= 13: 0.93/100000 (1 month) |
| Male, with suicide attempt | Age < 13: 0.16/100000 (1 month)  Age >= 13: 1.24/100000 (1 month) |
| CDRS-R score monthly change ($\beta_{s_{ij}}$)  Equations (10) and (11) | Mean (variance) |
| $\beta_{s_{ij}}$ in months with treatment |  |
| Non-treatment resistant | High response: -9.46 (3.06); Late response: -4.76 (3.06); Limited response: -3.53 (3.06) |
| Treatment resistant | High response: -4.98 (3.06); Late response: -5.14 (3.06); Limited response: -3.37 (3.06) |
| $\beta_{s_{ij}}$ in months without treatment | -1.84 (3.06) |

**Appendix Table 3 Parameters Adjusted to Simulate Different Intervention Scenarios**

| **Intervention** | | **Adjusted Parameter** |
| --- | --- | --- |
| Intervention 1 | Depression screening and have screened-positive ones initiate treatment | Equation (4) $\beta_{t0}$ |
| Intervention 2 | Reducing dropping out during treatment for depression | Equation (7) $\beta_{drop0}$ |
| Intervention 3 | Suicide screening and treatment among the depressed individuals | Individuals’ probability of suicide attempt and probability of suicide |
| Intervention 4 | Suicide screening and treatment in all medical care settings | Individuals’ probability of suicide attempt and probability of suicide |

**References**

1. Ulla Timlin, Helinä Hakko, Raija Heino, Helvi Kyngäs. Factors that Affect Adolescent Adherence to Mental Health and Psychiatric Treatment: a Systematic Integrative Review of the Literature. *Scand J Child Adolesc Psychiatry Psychol*. 2015;3(2). doi:10.21307/sjcapp-2015-010

2. A Double-blind, Efficacy and Safety Study of Duloxetine Hydrochloride Versus Placebo in the Treatment of Japanese Children an Adolescents With Depressive Disorder.

3. Michael G. Koelch, Ferdinand Keller, Nina Sproeber, et al. Convergence of children´s depression rating scale-revised scores and clinical diagnosis in rating adolescent depressive symptomatology. *Ment Illn*. 2012;4(1):e7-e7. doi:10.4081/mi.2012.e7

4. Zhang C, Zafari Z, Slejko JF, Castillo WC, Reeves GM, dosReis S. Impact of Undertreatment of Depression on Suicide Risk Among Children and Adolescents with Major Depressive Disorder: A Microsimulation Study. *Am J Epidemiol*. Published online January 27, 2023:kwad022. doi:10.1093/aje/kwad022

5. Fontanella CA, Bridge JA, Marcus SC, Campo JV. Factors associated with antidepressant adherence for medicaid-enrolled children and adolescents. *Ann Pharmacother*. 2011;45(7-8):898-909. doi:10.1345/aph.1q020

6. Melhem NM, Porta G, Oquendo MA, et al. Severity and variability of depression symptoms predicting suicide attempt in high-risk individuals. *JAMA Psychiatry*. 2019;76(6):603-613. doi:10.1001/jamapsychiatry.2018.4513

**Appendix Figure 1. An Illustration of the Microsimulation Model Process**


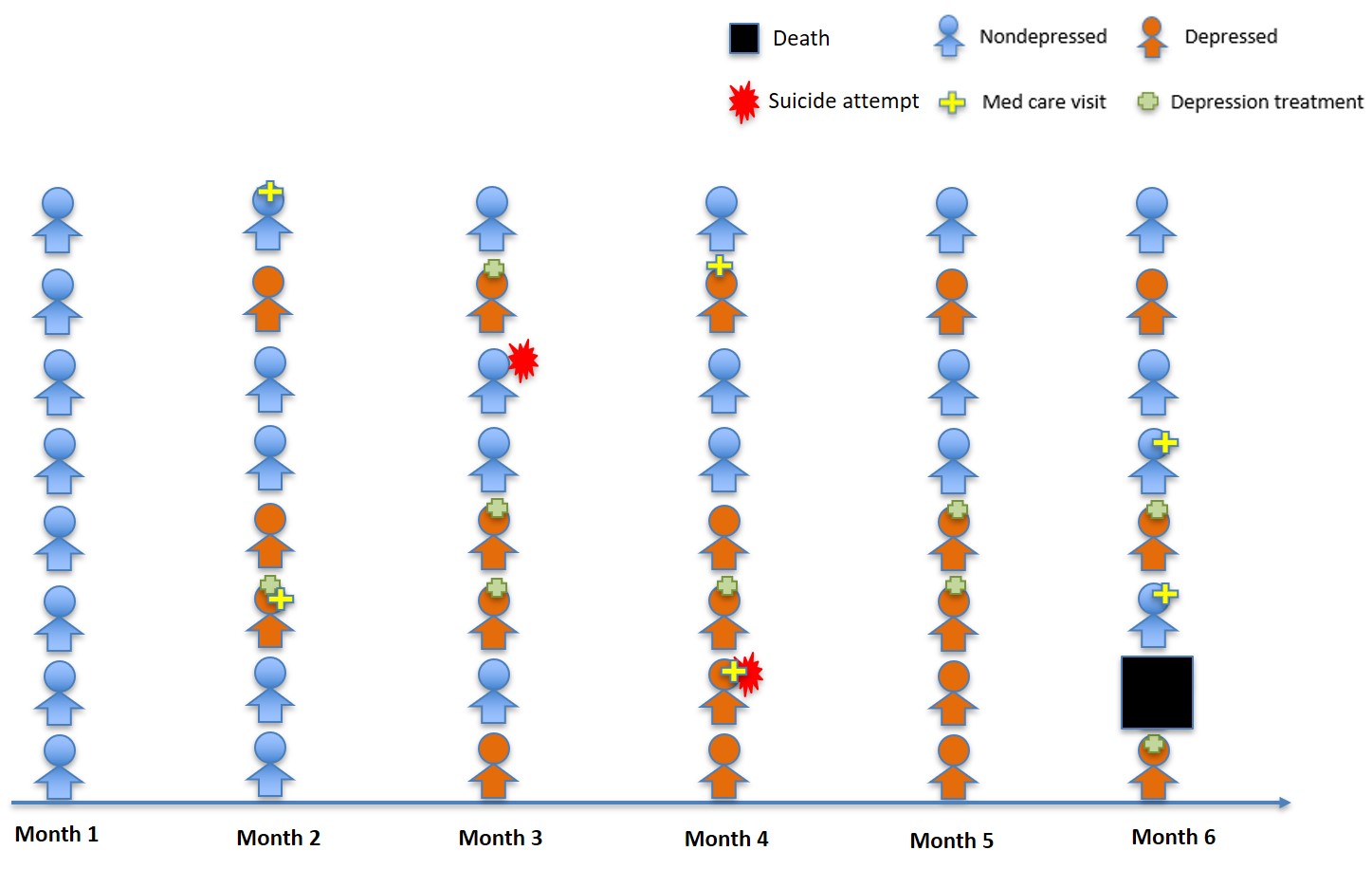

Supplement: Supplementary file 1 [file Data_Sheet_1.docx]
